# Efficacy of botulinum toxin A for the treatment of hemiparesis in adults with chronic upper limb spasticity

**DOI:** 10.11604/pamj.2020.35.55.16091

**Published:** 2020-02-25

**Authors:** Sameh Ghroubi, Samar Alila, Wafa Elleuch, Houda Ben Ayed, Chokri Mhiri, Mohamed Habib Elleuch

**Affiliations:** 1 Department of Physical Medicine and Rehabilitation, University Hospital Hbib Bourguiba, Sfax, Tunisia; 2 Department of Epidemiology and Community Medicine, University Hospital Hedi Cheker Sfax, Tunisia; 3 Department of Neurology, university hospital Hbib Bourguiba Sfax Tunisia

**Keywords:** Botulinum toxin-A, function, stroke, upper limb

## Abstract

**Introduction:**

this study aimed to evaluate the effectiveness of botulinum toxin A (BoNT-A) injection in hemiparetic patients with chronic spasticity in the upper limb resulting from stroke or traumatic brain injury.

**Methods:**

we conducted a retrospective study including 45 patients seen, in our department of Physical Medicine and Rehabilitation, between January 2014 and December 2016. All patients received an injection of BoNT-A (Dysport, 100 U/ml). Affected upper-extremity muscles could be injected as per the investigator’s discretion to a maximum total dose of 1000 U. We evaluated muscle tone using Modified Ashworth Scale (MAS). Functional disability was assessed using Modified Frenchay Scale (MFS), Nine Hole Peg Test (NHPT) and Barthel Index (BI). Quality of life (QoL) was assessed using the 36-Item Short Form Health Survey (SF-36). The achievement of treatment goal was assessed by the Goal Attainment Scaling (GAS).

**Results:**

patients decreased their MAS score over the first and the third months (p<0.05). MFS showed improvement at 1 month after injection with a median change from baseline of 8 (range: 1-16; p<0.001). The change from baseline ranged from 0 to 5 points for NHPT at 1 month after injection (p< 0.001). This functional improvement was maintained to 3 months. Improvements in Barthel Index was observed at 3 months with a median change from baseline of 5 points (range 0-15; p<0.001). The mean change from baseline of SF-36 score was 4.77 ± 3.39 (p<0.001). The mean GAS T-score was 47.04 ±7.78 (median 50, IQR 7.7), giving a mean (SD) change from baseline of 25.36 ± 8.46 (95% CI 22.82 to 27.90; p <0.001). Binary logistic regression was used to identify the independent factors predicting a favorable functional outcome of Bon-T treatment. It showed that neglect was independent predictive factor treatment failure (p=0.009, OR=3.2) while previous injection of BoNT-A was an independent predictive factors of treatment success (p=0.009, OR=0.3).

**Conclusion:**

our study showed a good response to BoNT-A injection delivered in the management of chronic upper limb spasticity resulting from stroke or traumatic brain injury. It demonstrated its outcome in improving muscle tone, function and QoL. It also showed that the majority of patients achieved their goal as defined at the start of the treatment, mainly for patients who received previous injection of BoNT-A.

## Introduction

Upper limb spasticity may cause deformity, pain and reduced function [1]. The treatments of this spasticity include oral antispastic drugs, botulinum toxin and surgical treatments as selective neurotomy and orthopedic surgery [1]. The botulinum toxin is increasingly used to treat spasticity due to stroke or traumatic brain injury (TBI). This treatment has been widely shown to reduce muscle tone [2] and improve basic upper limb activities such as hand hygiene and facilitation of dressing [3]. However, measuring the effectiveness of BoNT-A treatment is challenging in the context of upper limb spasticity because of large diversity in terms of patient presentation, potential for rehabilitation and goals for treatment. Current guidelines for BoNT-A use in the management of spasticity advocate the application of focused outcome evaluations, targeted on the attainment of goals that are both relevant to the treatment intentions and important to the individual [1]. Cohort study design in this context must therefore be flexible enough to consider variation in the individual clinical presentation and the nature of the goals set for each patient [4]. In addition, functional change has been harder to demonstrate, particularly where impact on active function is limited by underlying motor dysfunction [5]. Despite the large debate in the literature regarding the effectiveness of botulinum toxin in improving active function, very few studies have sought to identify factors predicting a favorable functional outcome of this treatment in hemiparetic patients. This current study aimed to evaluate, with a person-centred measure of outcome, the effectiveness of BoNT-A injection, particularly in the active function, in hemiparetic patients with disabling spasticity in the upper limb resulting from chronic stroke or traumatic brain injury, and to identify baseline factors that predict favorable functional outcome of this treatment.

## Methods

**Study design and participants:** we conducted a retrospective study including patients with hemiparesisthat were reviewed in our Physical Medicine and Rehabilitation Department, between January 2014 and December 2016. The main eligibility criteria for the inclusion of patients were adults ≥ 18 years with hemiparesis for at least 6 months after a stroke or traumatic brain injury (TBI), and whose a Modified Ashworth Scale (MAS) score in target muscle group was at least 2. Agreement on achievable goals set was also required. The exclusion criteria were any medical disorder increasing the risk of botulinum-toxin-A-related adverse events; severe muscle atrophy or infection in target sites, or current anticoagulant therapy with INR ≥ 3.5. Women were excluded if they were pregnant or planning to become pregnant during the course of the study. Patients were not excluded if they received previous BoNT-A injections. All patients signed approved informed consent prior to undergoing any study-specific procedures. The study was approved by the regional ethics committee (Ref: CCP Sud: 0043/2017).

**Study treatment:** all patients received an injection of botulinum toxin type A (100 U/ml). Affected upper-extremity muscles, from the shoulder to fingers, could be injected as per the investigator’s discretion, based on the participant’s disability to a maximum total dose of 1000 U. The dosage of toxin for each muscle was based on muscle size and degree of increased tone in each specific muscle. Injections employed a needle stimulation technique, with a monopolar injection electrode. Once the target muscle was identified, by obtaining an appropriate contraction with the lowest possible stimulus intensity, BoNT was injected into one to four sites, based on the size of the muscle. Immediately after injection, we prescribed a physiotherapy and occupational therapy program which included 3 sessions per week for 6 months. Patient’s adherence to this program was recorded throughout the study. To carry out on days they were not attending therapy, participants were also given a home exercise program which included prolonged self-administered stretch postures and rapid alternating movements. Antispastic medication had to be maintained at a stable dose during the study.

**Outcome measure:** clinical examination included the evaluation of the muscle tone by MAS [6], passive and active range of motion (ROM) in shoulder, elbow, wrist and fingers, and pain using Visual Analogue Scale (VAS). Functional disability and activity limitation of upper limb were assessed using: modified Frenchay Scale (MFS) which is based on a masked video review of ten everyday living tasks rated on a 10-point visual analogue scale. Six bimanual and four unimanual tasks were to be done with the paretic hand [7]; the Nine Hole Peg Test (NHPT) which measure fine manual dexterity [8]; the Barthel Index (BI) which was used as a more general assessment of activities of dayling life [9]. Quality of life was assessed using the 36-Item Short Form Health Survey (SF-36) [10]. The achievement of treatment goal was assessed by the Goal Attainment Scaling (GAS). We used the simplified GAS approach described by Tuner- Strokes [11], which is based on the original method described by Kiresuk and Sherman [12] but designed to be timely and practical for use in a busy clinic setting. GAS results were expressed by two different ways: scoring each goal between -2 and +2, resulting in as many raw scores as there are scales [13] and giving a direct result for each goal, which is easily understood by the patient and easy to use in clinical practice: : “+2” is the initial pretreatment (baseline) level, “1” represents progression towards the goal without goal attainment, “0” is the expected level after treatment, (and therefore, the “most likely” level after treatment), “+1” represents a better outcome than expected, and “+2” is the best possible outcome that could have been expected for this goal; a T-score [11], which is supposed to enable GAS scores to be normalized and then analyzed with parametric statistics. Since all the goals have the same weight (difficulty and importance), we used the simplified equation of T-score [14]:

$$n=\frac{z_{\infty }^{2}pq}{d^{2}}$$

Where: Xi = the GAS score; C = a coefficient that depends on the patient’s number of scales (and thus the number of scores). All assessments were done at baseline (screening visit) and at 1, 3 and 6 months (follow-up visits), except those of BI and GAS which were recorded at baseline and at 3 months and the SF-36 which was done at baseline and at the end-of-study visit (at 6 months).

**Statistical analysis:** all statistical analysis were conducted with SPSS.20 software package. Baseline characteristics were presented as descriptive statistics. Mean and SD were reported for data that fulfilled the criteria for normal distribution. Data following a non-normal distribution were described by median and range (minimum and maximum). Participants” treatment response was calculated as the change in scale from baseline to follow-up. Comparison between mean scores before and after the treatment was analyzed using a parametric (paired t-test) or nonparametric (Wilcoxon signed ranks) test, depending on the type of data being analyzed. Scores of ‘1+’ were entered as 1.5 in the calculation of MAS scores. Baseline scores of GAS were rated as −1=‘some function’ and -2=‘no function’ with respect to the goal. ‘Responders’ were those who achieved their goal (GAS score 0, 1 or 2: goal achieved). Relationships between scores were examined using Spearman’s rank correlation coefficients. To identify the independent factors predicting a favorable functional outcome of BoN-T treatment, binary logistic regression test was performed using the variables showing statistically significant differences in the univariate analysis of the measured factors. A p value of 0.05 was used for statistical significance.

## Results

**Characteristics of participants:** this study included 45 patients. They were referred from the neurological service (68.9%), the intensive care unit (ICU) (15.6%), and the orthopedic service (15.6%). Only patients from ICU were sent for botulinum toxin injection. The other patients were sent only for rehabilitation care and the botulinum toxin injection was indicated by a consultant in rehabilitation medicine and then discussed in our neuro-orthopaedic staff. The mean age was 50.29±14.8 years and the mean time since onset of incident was 3.28 ± 3.01 years. Sixty-two per cent of the population was men; 75.55 % had stroke and 24.44 % had traumatic brain injury. Left and right hemisphere localization was approximately equal (55.6% and 44.4%, respectively). Baseline characteristics are detailed in Table 1.

**Table 1:** Baseline clinical characteristics of the population

Parameter	Values
**Cause of spasticity, n (%)**	
Stroke	34 (75.55)
TBI	11 (24.44)
**Spasticity (MAS) of the most frequently injected muscle in each joint, median [min-max]**	
Shoulder: Pectoralis major	3 [2-3]
Elbow: Biceps brachii	3 [2-3]
Wrist: Flexor Carpi	3 [2-3]
Fingers: Flexor digit. Superficialis	3 [2-4]
**Others symptoms, which may impact on functional outcome, n (%)**	
Communicative impairment	12 (26.6)
Cognitive impairment	9 (36)
Spatial neglect	6 (13.3)
Osteoma of upper limb	7 (15.6)
Sensory	15 (33.33)
**MFS, median [min-max]**	20 [5-38]
**NHPT, median [min-max]**	0 [0-3]
**BI, median [min-max]**	35[25-75]
**SF-36, mean ± SD**	
PCS	40.57 ± 13.86
MCS	41.34 ± 13.53
Total	40.41 ± 12.9
**GAS T-score, mean ± SD**	21.68 ± 2.06

TBI: Traumatic brain injury, MAS: modified Ashworth Scale, MFS: modified Frenchay Scale, NHPT: Nine Hole Peg Test, BI: Barthel Index, PCS: Physical Composite summary, MCS: Mental Composite summary; GAS: Goal Attainment Scale

### Treatment

**BoNT-A injection history:** twenty-six participants (57.8 %) had received previous injection of BoNT-A in the upper limb. The median time since the last injection was 12 months (IQR 0; range 6-36 months). The median time since the first injection was 24 months (IQR 2.25; range12-84 months). The median number of BoNT-A injections previously received by the participants was 2 (IQR 2; range 1-11).

**BoNT-A treatment:** in this cohort, there was very wide variation in the total dose of BoNT-A. The most commonly injected muscles and doses are shown in Table 2. The most frequently injected muscles were the Flexor carpi (84.44%), followed by Flexor digitorum Superficialis (71.11%), Pronator teres (51.11%) and biceps (46.66%). With the exception of the pectoralis major (which was injected in 13.3% of patients), the shoulder muscles were relatively rarely injected. Multiple injection points were most commonly used in the larger and more proximal muscles, such as the biceps (85%) and the pectoralis major (66.7%).

**Table 2:** Most commonly injected muscles and technique within each upper limb segment

Group/muscle	Total number injected: n (%)	Number of units: Median [Min-Max]	Multiple Points: n(%)
Shoulder			
Deltoideus	1 (2 %)	240	1 (100%)
Subscapularis	1 (2 %)	150	0
Pectoralis major	6 (13.3 %)	150 [50-300]	4 (66.7%)
Latissimus dorsi	1 (2 %)	200	0
Upper arm			
Biceps brachii	21 (46.66 %)	200 [50-400]	17 (85%)
Brachialis	1 (2 %)	100	0
Triceps brachii	1 (2 %)	170 [100-240]	
Lower arm			
Flexor carpi ulnaris	38 (84.44 %)	100 [50-150]	7 (19.4%)
Flexor carpi radialis	38 (84.44 %)	100 [60-250]	2 (5.3 %)
Flexor pollicis longus	15 (33.33 %)	50 [20-250]	0
Pronator teres	23 (51.11 %)	100 [50-180]	0
Square pronator	5 (11.11 %)	100 [60-150]	0
Brachioradialis	12 (26.66 %)	100 [60-300]	1 (9.1 %)
Flexor digit. Superficialis	32 (71.11 %)	150 [60-400]	17 (54.8%)
Flexor digit. Profondus	7 (15.55 %)	100 [50-160]	2 (33.4%)
Hand/fingers			
Adductor pollicis	19 (42.22 %)	50 [20-80]	0
Interossei dorsales	5 (11.11 %)	100 [50-100]	0

Percentages are based on the number of participants injected in the muscle

**Concomitant treatments:** in follow-up visit, over two-thirds (71.1%) of patients were adherent to physiotherapy prescribed at the start of the study. Only 57.8 % of participants received occupational therapy and 55.6% practiced home exercises. The proportion of patients on antispasmodic medication was 44.4%.

### Evolution of outcome measure

**Spasticity:** patients decreased their MAS score for shoulder, elbow, wrist and fingers over the first month. The change from baseline was 0.66±0.41; 0.95±0.46; 1±0.65; and 1.03±0.59 points for pectoralis major, biceps brachii, flexor carpi ulnaris, and flexor digit superficialis respectively (p<.001). This tone reduction was maintained over the third month (p<.001) but not statistically significant 6 months after injection (Table 3).

**Table 3:** Change from baseline for outcome measures including spasticity, range of motion, pain, functional outcome, quality of life and GAS T score

	1 month			3 month		6 month
**Spasticity (MAS) of the most frequently injected muscle in each joint, median [min-max]**						
Shoulder: Pectoralis major	1[0-1]	p=.04	1[0-1]	p=.04	0[0-1]	p=.15
Elbow: Biceps brachii	1[0-2]	p<.001	1[0-2]	p<.001	0[0-1]	p=.05
Wrist: Flexor Carpi	1[0-2]	p<.001	1[0-2]	p<.001	0[0-1]	p=.06
Fingers: Flexor digit superficialis	1[0-2]	p<.001	1[0-1]	p<.001	1[0-1]	p=.08
**Passive Range of motions; mean ± SD**						
Shoulder	6.66 ±5.16	p=.001	6.1 ±4.91	p=.001	2.5 ±2.73	p=.05
Elbow	15 ±7.07	p<.001	14 ±3.02	p<.001	11 ± 8.48	p<.001
Wrist	15 ±14.14	p<.001	11 ±7.07	p<.001	7 ± 5.07	p<.001
Fingers	20 ±14.14	p<.001	17.5 ±1.6	p<.001	15 ±7.07	p<.001
**Active Range of motions; mean ± SD**						
Shoulder	8.33 ±7.52	p=.001	7.5 ±6.12	p=.001	5 ± 4.74	p=.001
Elbow	14.5 ±7.58	p<.001	12.5 ±7.58	p<.001	1.66 ± 8.89	p<.001
Wrist	20 ±14.14	p<.001	18 ±12.5	p<.001	17.5 ±1.6	p<.001
Fingers	15 ±7.62	p<.001	12.5 ±3.53	p<.001	7.5 ± 3.53	p<.001
**EVA Pain; median [min-max]**	1 [0-4]	p<.001	0[0-2]	p<.001	0 [0-1]	p=.08
**MFS; median[min-max]**	8 [1-16]	P<.001	4.5 [0-11.5]	p<.001	.5 [0-3]	p=.05
**NHP; median [min-max]**	0 [0-5]	p<.001	0 [0-6]	p<.001	0 [0-3]	p=.05
**BI; ; median [min-max]**			5[0-15]	P<.001		
**SF-36; mean ± SD**						
PCS					4.98 ±3.47	p<.001
MCS					4.53 ±3.71	p<.001
Total					4.77 ±3.39	p<.001
**GAS T score; mean ± SD**			25.36 ± 8.45	p<.001		

TBI: Traumatic Brain Injury, MAS: Modified Ashworth Scale, MFS: Modified Frenchay Scale, NHPT: Nine Hole Peg Test, BI: Barthel Index, PCS: Physical Composite summary, MCS: Mental Composite summary; GAS: Goal Attainment Scale

**Range of motion:** there was a significant improvement in the passive and active ROM for shoulder, elbow, wrist and finger extension at 1, 3 and even 6 months after injection (p<.001).

**Pain:** there was a significant decrease in pain VAS score from baseline at 1 and 3 months after injection (p<.001). The change was not significant at 6 months.

### Functional Outcome

**Modified Frenchay Scale:** this functional measure showed improvement at 1 month after injection with a median change of 8 points from baseline (range: 1-16; p<.001). This improvement was maintained at 3 months after injection with a 4.5 median change from baseline (range: 0-11.5; p<.001), and then decreased at 6 months (p> .05). The change from baseline for bimanual activities of MFS at 1 month were: pick up knife and fork: 0.95 ± 0.63 point; open and close jam jar: 0.87±0.58 point; put toothpaste on tooth brush: 0.96 ±0.59 point; rule line with ruler: 0.8±0.62 point, clip 3 clothe-pins on paper pad edge: 0.61±0.62 point, sweep floor with broom: 0.78±0.6. The change from baseline for unimanual activities were: pick up big bottle: 0.78±0.51 point, pick up small bottle 0.81±0.55 point, pick up comb and mimic combing: 0.58 ±0.43 point; pick up glass and bring it to mouth: 0.67±0.47 point.

**Nine hole peg test:** active upper limb function as assessed by NHP test was significantly improved. The change from baseline ranged from 0 to 5 points for NHPT at 1 month after injection (p< .001). However, there was a larger change from baseline at 3 months (median: 0; range: 0-6; p<.001). This improvement decreased and wasn’t statistically significant 6 months after injection.

**Barthel index:** Improvements in Barthel Index was observed at 3 months with a median change from baseline of 5 points (range 0-15; p<.001).

**Quality of life:** we noted a significant improvement in patients’ quality of life. The mean change from baseline of total SF-36 score was 4.77 ± 3.39 (p<.001). Physical and mental component summary of the SF-36 showed a statistical change at the end of the study (4.98±3.47; 4.53±3.71; respectively, p<.001).

**Goal attainment scale:** goal areas set at baseline are shown in Table 4. Goals were most commonly set in the areas of passive function (55.82 %) followed by active function (26.46%) and pain (17.64%). Overall, 126 (74.11%) goals were achieved (or overachieved). Although the rate of achievement was lower for active function goals in comparison with passive function goals, in this series, a total of 45 goals were set in relation to active function of which 27 (60%) were achieved, either as expected (20 (44.4%)) or beyond expectation (7 (15.55%)). Pain reduction was a goal for treatment in nearly two-third of the patients and was achieved in 90%. At follow-up, the mean (SD) GAS T-score was 47.04 (7.78; median 50, IQR 7.7), giving a mean (SD) change from baseline of 25.36 (8.46; 95% CI 22.82 to 27.90; p <.001).

**Table 4:** Goal attainment scale: goal areas and achievement rates

Goal area	Sub categories	Goal Set n(%)	Not achieved n(%)	Partially achieved n(%)	Goal achieved n(%)
**Pain**	Pain	30 (17.64%)	0	3 (10 %)	27 (90%)
**Passive function**	Positioning the limb	39 (22.94%)	3 (7.7%)	5 (12.8%)	31 (79.5 %)
	Hygiene of hand	27 (15.88%)	1 (2.2%)	7 (15.6 %)	19 (70,4%)
	Dressing	29 (17%)	4 (13.8%)	3 (1.3%)	22 (75.8)
**Active function**	Holding/bimanual function	32 (18.82%)	2 (6.3%)	10 (31.3%)	20 (62.5%)
	Fine motor/dexerity	13 (7.64%)	0	6 (46.2%)	7 (53.9%)
**Total**		170	10 (5.88%)	34 (20%)	126 (74.11%)

**Correlations:** there was a significant correlation between the baseline score and the change from baseline after BoNT-A injection for MFS (p <.001; rho = 0.524) and NHPT (p = .001; rho = 0.478). GAS T-score correlated, moderately, with the change from baseline of VAS pain score (Spearman rho= 0.349; p=.01) and of BI score (Spearman rho 0.349; p=.01). It correlated more strongly, however, with the gain in total MFS score (Spearman rho 0.647; p<.001) and in NHPT score at follow-up (Spearman rho 0.424; p=.004).

**Identification of factors predicting a favorable functional outcome:** in univariate analysis, previous injection of BoNT-A (p=.006) and occupational therapy (p=.02) were associated with favorable functional response to BoNT-A treatment, however spatial neglect (p=.01) and cognitive impairment (p=.04) were associated with treatment failure. Functional outcomes were not associated with sensory, osteoma and communicative impairment. In multivariate analysis, spatial neglect was an independent predictive factor of BoNT-A treatment failure (p=.005, OR=6.876) while previous injection of BoNT-A was an independent predictive factor of treatment success (p=.003, OR=0.29).

## Discussion

This study showed a good response to BoNT-A injection delivered in the management of chronic upper limb spasticity in patients with hemiparesis for nearly 3 years after a stroke or TBI. It demonstrated its outcome in improving muscle tone, passive and active function and quality of life. It also showed that the majority of patients achieved their goal as defined at the start of the treatment, mainly for patients who received previous injection of BoNT-A and who practiced occupational therapy. BoNT-A reduced muscle tone and the level of reduction was similar to previous studies [15, 16]. The peak efficiency was at 1 month after injection and the improvement was not sustained at 6 months. It is not surprising that antispastic effect wears off after 6 months. In fact, BoNT-A prevents the release of the neurotransmitter acetylcholine from axon endings and this blockage is slowly reversed as the toxin loses activity [17]. Besides, and like others reports, we found improvements occurred in the active and passive range of motion [18]. The improvement of active ROM suggests that the co-contraction was an important component of reduced upper limb ROM. Botulinum toxin type A was demonstrated to have a benefit in terms of pain reduction in which decreased pain VAS was seen at 1 and 3 months, but not at 6 months. The effect on reducing pain may be through a mechanism of spasticity reduction since muscle tone was not decreased at 6 months. Two previous trials had shown that botulinum toxin reduces upper limb pain up to 6 and even to 12 months [19, 20] and explained this finding by the avoidance of complications of spasticity such as spasm and contracture or by a direct analgesic effect by blocking transmission of neurotransmitters involved in pain pathways. More important was the functional outcome which was significant in our study and consistent with previous reports [21–25]. Despite spasticity is believed to contribute to reduce active function, the precise relationship between spasticity and motor performance is debated [25, 26]. Although there are those that advocate spasticity as an important component of reduced upper limb function, others believe the main problem is motor weakness [20]. Because this study demonstrated improved active function, it supported the argument that spasticity is of paramount importance. However detecting the functional effectiveness of BoNT-A treatment and comparing different studies is generally difficult due to the various outcome measures used by investigators [27]. In order to establish the best outcome measures, our study used the MFS which has six bimanual tasks among ten [28]. In fact, bimanual activities seem to be a good test for assessing progress in this type of population, they are more sensitive to change and therefore more relevant to assess functional improvement of spastic upper limb [27, 29]. This score showed improvement at 1 and 3 months after injection and as described by Ferrapie and Slawek [27, 29], there was a best impact of BoNT-A in bimanual activities. Concerning fine manual dexterity assessed by NHPT, we found significant improvement like others studies [24, 25, 30]. The change from baseline at 3 months after injection was larger than the change at 1 month, which may be due to muscle weakness at the time of maximum BoNT-A effect.

Additionally, there was a significant correlation between the baseline score and the change from baseline after BoNT-A injection for MFS and NHPT. This suggests that functional improvement was less likely to occur for patients with severe initial impairment. A statically significant improvement was also demonstrated for Barthel Index at 3 months. However, the majority of studies didn’t show any improvement in this scale [31].In fact, the rating of this scale is based on whether the patient is dependant, requires help or independent, and at the screening visit, a large number of patients affirmed that they often received help even when they didn’t need it. This may be explained by the fact that in our society, family is of paramount importance and its members are the most of the time willing to help each other. Since the first visit, we explained to both patients and their helpers the importance of self-reliance for amelioration and then we encouraged patients to be more independent by trying to accomplish the activities of daily life without external assistance. Regarding quality of life, we recorded a significant improvement at 6 months after botulinum toxin injection like others studies [21, 32, 33]. Although the anti-spastic effect of the toxin wearied off at 6 months, the benefits on quality of life were sustained. This longer-term effect was likely a result of the therapy and the interaction with the therapist. In fact, the intramuscular injection of Botulinum toxin facilitated stretching which become less painful and then facilitated rehabilitation exercises. This observable improvement by the participants in their therapy program is a life-positive change that can affect well-being and motivation. This change can be also attributed to the high cost of the Botulinum toxin which makes our patients more optimistic, believe more on its benefits, and subsequently become more adherent to the therapy to reach maximum effect and justify investment. In order to evaluate the benefits of treatment in terms of the attainment of individual person-centered goals, we chose the Goal Attainment Scale. This scale enables involving patient and his family in the treatment project and provides important insight into the nature of goals that are chosen as priorities for treatment and those that are most likely to be achieved [5]. Almost 73% of patients achieved their goal, as defined by the patients, together with their clinical team, at the start of treatment. The most commonly achieved goal area was pain (set in 30 patients), where attainment was around 90 %. Perhaps the achievement rate of active function was somewhat lower compared to other goals, nevertheless, more than half of patients (56.25 %) achieved their active function goals.

Over and above the attainment of each goal, the GAS T-score provides an overall assimilation of attainment goals regardless of the number of goals set. Some authors have cast doubt on the value of calculating a GAS T-score [34]. In this study, we put considerable efforts to ensure that GAS was applied rigorously, so it is worth reflecting on the added value of the GAS T-score in this context. If goals are set in an unbiased fashion, and are neither overambitious nor overcautious, the mean GAS T-score should be around 50 (±SD 10) [34]. Our mean (SD) GAS T-score at follow-up of 47.04 (7.78) provided a useful quality check of the team’s ability to set and negotiate achievable goals, neither overestimating nor underestimating the expected outcome. Besides, GAS T-scores provide a single numerical evaluation of overall goal achievement for comparison with other outcome measures [34]. There were significant correlations between GAS T-score and others scales (NHP, MFS, BI, VAS pain) which provide further support for GAS as a meaningful person centered measure of outcome in this context. Then, its use enables setting precise goals including various domains, which may replace other standardized measures. In this study, we looked for prognostic factors of achievement of active function goals within the baseline data set. Results indicated that spatial neglect impairment was independent predictive factor of treatment failure. This was consistent with the report of Turner-Stokes [34]. Cognitive impairment was also associated with functional response to BoNT-A treatment in univariate analysis. The most likely explanation for this is that patients with cognitive impairment are more likely to have difficulty complying with any treatment program. Previous injection of BoNT-A was an independent predictive factor of treatment success. Possible explanations for this may be that physicians optimized muscle selection and dose-ranging through previous injections, and also patients benefited from their previous experience to choose their treatment goals. This finding highlights the benefits from the longer term treatment by botulinum toxin of upper limb spasticity and suggests that patients continue to receive improvement from repeated injections. Besides, in our study, occupational therapy was associated with favorable functional response to BoNT-A injection in univariate analysis. Although occupational therapy is often recommended to address upper limb impairment and difficulty with daily life activities, only 60% of our patients had received this therapy since it is not covered by the national health insurance and the number of occupational therapist was limited in our country. Despite these outcomes, this study had some limitations which included the small group, the assessment of only one injection session and the absence of placebo injection. However, there were several strengths in this study. It provides useful information about the way BoNT-A is used in clinical practice in our country, and demonstrates that its effectiveness in the management of chronic spasticity can be documented using individual person-centred goals. Moreover, a large majority of the patients achieved their treatment goals in terms of passive and active functions, demonstrating that BoNT-A injections contribute to an improvement in the daily life activities.

## Conclusion

Treatment of chronic upper limb spasticity in patients with hemiparesis after a stroke or TBI with BoNT-A injections is a valuable and useful method of treatment for improving spasticity, ROM, pain, passive and active function and quality of life. The goal of treatment should be clearly established prior to BoNT-A therapy, because functional improvement was unlikely demonstrable for patients with spatial neglect or cognitive impairment. However, it may be demonstrable mainly in patients who received previous injection of BTX-A and who practiced occupational therapy. Further studies are required, on one hand for better assessment of repeated injections of botulinum toxin, on the other hand at earlier phases of the disease.

### What is known about this topic

- Upper limb spasticity may cause deformity, pain and reduced function;
- Botulinum toxin is increasingly used to treat spasticity due to stroke or traumatic brain injury;
- Botulinum toxin has been widely shown to reduce muscle tone and improve basic upper limb activities such as hand hygiene and facilitation of dressing.

### What this study adds

- This study demonstrated the effectiveness of botulinum toxin in improving active function of spastic upper limb due to stroke or traumatic brain injury;
- This study demonstrated the effectiveness of Botulinum toxin by focusing on the attainment of goals that are both relevant to the treatment intentions and important to the individual. Therefore, this study considered variation in the individual clinical presentation and the nature of the goals set for each patient;
- This study identified, in a multivariate analysis, factors predicting favorable functional outcome of botulinum toxin treatment in hemiparetic patients.

## Competing interests

The authors declare no competing interests.
